# Synthesis of Hydrophobic Derivatives of Stilbenes with Improved Permeability and Limited Phase II Reactions

**DOI:** 10.1021/acs.jafc.6c00922

**Published:** 2026-04-15

**Authors:** Silvia Navarro-Orcajada, Irene Conesa, Francisco José Vidal-Sánchez, Adrián Matencio, José Manuel López-Nicolás

**Affiliations:** † Departamento de Bioquímica y Biología Molecular-A, Facultad de Biología, 16751 Universidad de Murcia — Regional Campus of International Excellence “*Campus Mare Nostrum*”, E-30100 Murcia, Spain; ‡ Department of Chemistry, University of Turin, via P. Giuria 7, 10125 Turin, Italy

**Keywords:** stilbenes, resveratrol, lipophilic, bioavailability, permeability, metabolism

## Abstract

Stilbenes can promote human health due to their several bioactivities. However, their low bioavailability is a limiting factor for the *in vivo* bioactivity of these plant bioactive compounds. This low bioavailability has been previously associated with their rapid absorption and phase II metabolism. In this work, the synthesis of hydrophobic stilbene derivatives is explored as a promising alternative. Chemical and enzymatic syntheses were compared, achieving higher efficiency with the former. The synthesized derivatives were characterized to ease future research. Hydrophobic resveratrol derivatives improved the apparent permeability of resveratrol across the Caco-2 intestinal cells. Moreover, hydrophobic derivatives of resveratrol and oxyresveratrol showed a lower degree of sulfation and glucuronidation compared to original stilbenes, which could be an advantage in avoiding phase II reactions that decrease the bioavailability of stilbenes. These results support hydrophobic stilbene derivatives as precursors of natural stilbenes, highlighting their strong potential for oral administration through functional foods in humans.

## Graphical abstract

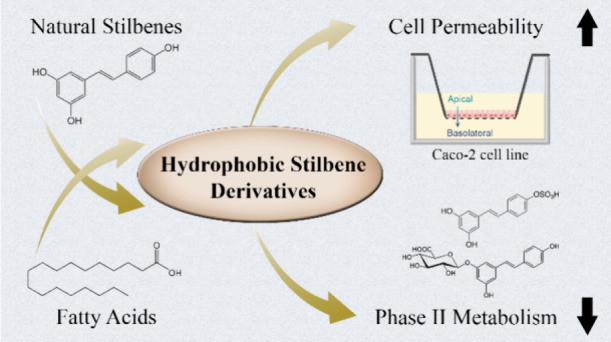

## 1 Introduction

Stilbenes are secondary metabolites from plants aimed to protect them from biotic and abiotic stress. They are naturally occurring in some botanical families such as Vitaceae (grapes), Pinaceae (pine), Ericaceae (blueberry), and Fabaceae (peanut), so they are ingested in small amounts through diet. This family of phenolic compounds can promote human health due to their antioxidant, anti-inflammatory, antimicrobial, cardioprotective, anticarcinogenic, and antiobesity activities, making them of great interest for the development of drugs, functional foods, and cosmetics.^1–8^

Among them, resveratrol (*trans*-3,4′,5-trihydroxystilbene) is the most extensively studied and cited stilbene, with over 32,000 publications in the Web of Science (2025). However, since the bioactivity of stilbenes is closely related to their structure, lesser-known analogues are claiming the spotlight because of their better physicochemical or biological properties.^5,9^ This is the case of pterostilbene (*trans*-3,5-dimethoxy-4′-hydroxystilbene), whose methoxy groups increase the molecule’s lipophilicity and stability, resulting in better oral bioavailability compared to resveratrol. Similarly, oxyresveratrol (*trans*-2,3′,4,5′-tetrahydroxystilbene) and piceatannol (*trans*-3,3′,4′,5-tetrahydroxystilbene), which possess an additional hydroxyl group, exhibit higher antioxidant potential than resveratrol.^10,11^

Nevertheless, the low bioavailability of stilbenes remains a limiting factor for the *in vivo* bioactivity of this family of bioactive compounds. This low bioavailability has been associated with their rapid absorption and phase II metabolism that generates more soluble conjugates (sulfates and glucuronides).^10^ These conjugated metabolites can be easily eliminated in the urine,^10^ and a fraction of them may also reach target tissues, contributing to *in vivo* activity through regeneration of the parent compound.^12,13^ In addition, the gut microbiota can also metabolize stilbenes, producing compounds such as dihydroresveratrol and lunularin in the case of resveratrol, which have also been shown to reach some tissues.^14^ Despite this, reported levels of free stilbene in target tissues remain low.^14^

Previous studies have shown that derivatization of bioactive compounds can help avoid phase II reactions, improving bioavailability.^15–17^ These derivatives can act as precursors of the original molecules, making their transport to target tissues easier.^18,19^ In addition, lipophilic derivatizations can bypass hepatic metabolism by transporting molecules through the lymphatic system.^20–23^ Therefore, lipophilic derivatization may offer two potential mechanisms to reduce metabolism of bioactive compounds and increase bioavailability: (1) by masking hydroxyl groups, esterification may hinder phase II enzymes from attaching glucuronide or sulfate moieties, potentially decreasing intestinal or enterocyte metabolism,^15,16^ and (2) by increasing lipophilicity, these derivatives may enter systemic circulation via lymphatic transport, thereby bypassing hepatic metabolism.^21,23^

Structural modifications that increase lipophilicity, however, can also cause problems in handling these molecules, as they are less soluble in aqueous matrices. The use of cyclodextrins (CDs) or hyperbranched CD-based polymers (HBCD-Pols) could alleviate this issue, as they are capable of improving solubility by encapsulating poorly water-soluble molecules in their slightly lipophilic cavity and, in the case of HBCD-Pols, also by surface adsorption in their polymer network.^4,24,25^

For all these reasons, the synthesis and characterization of novel hydrophobic stilbene derivatives were studied to avoid their biotransformation and promote their cellular uptake. Both chemical and enzymatic syntheses were investigated to find the optimal procedure to synthesize these molecules. Finally, the cell permeability and phase II metabolism of some hydrophobic stilbene derivatives were analyzed and compared to those of their original stilbenes to discover the potential advantages of these molecules as stilbene precursors.

## 2 Materials and Methods

### 2.1 Materials

All *trans*-stilbenes used (resveratrol (>99%), piceatannol (>98%), oxyresveratrol (>98%), and pterostilbene (>98%)) and *N*,*N*′-dicyclohexylcarbodiimide (DCC) were obtained from TCI (Zwijndrecht, Belgium). Fatty acids (stearic acid (>97%), palmitic acid (>98%), and oleic acid (>99%)), 4-dimethylaminopyridine (DMAP), vinyl stearate, Novozym 435 (≥5000 U/mg), dimethyl sulfoxide (DMSO) and *N*,*N*-dimethylformamide (DMF) were obtained from Sigma-Aldrich (Madrid, Spain). Chloroform (>99%), methanol (HPLC grade), acetonitrile (HPLC grade), and water (HPLC grade) were obtained from Fisher Scientific (Loughborough, United Kingdom). Other reagents were obtained from Merck/Sigma-Aldrich (Madrid, Spain).

### 2.2 Methods

#### 2.2.1 Synthesis and Purification of Hydrophobic Stilbene Derivatives

##### 2.2.1.1 Chemical Synthesis of Hydrophobic Stilbene Derivatives

Hydrophobic stilbene derivatives were synthesized chemically via Steglich esterification in anhydrous chloroform and DMF (94:6).^26^ In a three-neck flask equipped with a condenser, stilbenes (4.4 mM), fatty acids (4.4 mM), DCC (4.4 mM), and DMAP (44 μM) were added. The reaction was stirred gently at 25 °C in an inert atmosphere for 24 h.

The formation of products was monitored by thin-layer chromatography on silica plates using chloroform and methanol (9:1) as the mobile phase and by LC–MS on an Agilent HPLC 1200 series equipped with a time-of-flight (TOF) 6220 detector (acquisition range 100–1100) in negative mode using a mobile phase of 0.6 mL/min of methanol and water (60:40) with 20 mM ammonium acetate at 25 °C 5 min. The reaction yield was obtained from LC–MS analyses as the percentage of substrate converted, calculated based on the relative percentage of mass spectrometric signal.

##### 2.2.1.2 Enzymatic Synthesis of Hydrophobic Stilbene Derivatives

The enzymatic synthesis of resveratrol stearate was carried out using the enzyme Novozym 435 (immobilized lipase from *Candida antarctica*) based on the method described by Nicolosi et al. (2002) for resveratrol acetate synthesis with slight modifications.^27^ Reactions were conducted in a vial or flask with 2–10 mL of *tert*-amyl alcohol, 0.1 g of resveratrol, 0.268–1.34 g of vinyl stearate, and 0.1 g of Novozym 435 and were incubated in an inert atmosphere at 40 °C and 400 rpm for 90 h. The monitoring of product formation, as well as the calculation of the reaction yield, was performed as described above for the chemical synthesis.

##### 2.2.1.3 Purification of Hydrophobic Stilbene Derivatives

Purification was carried out by silica gel column chromatography using chloroform and methanol (9:1) as the mobile phase. Product elution was monitored by thin-layer chromatography on silica plates by using the same mobile phase.

Preparative HPLC purification was performed on a Shimadzu LC-20AP preparative HPLC system in reverse phase equipped with a DAD SPD-M40 detector, FRC-10A fraction collector (Shimadzu, Kyoto, Japan), and Kinetex C18 column of 5 μm (250 × 21.2 mm) (Phenomenex, Torrance, CA, USA). Injected samples were previously concentrated and eluted on Solid Purple C18 500 mg 3 mL columns (Análisis Vínicos, Tomelloso, Spain) with water and methanol. A flow rate of 25 mL/min at 25 °C was set with the following aqueous acetonitrile gradient: 40% from 0 to 2.5 min, 99% from 5 to 20 min, and 40% from 20.01 min. Detection and fraction collection were performed at 310 nm.

Fractions were collected and evaporated. After purification, the samples were stored in darkness at −20 °C. Care was always taken to protect them from direct exposure to light during their use.

#### 2.2.2 Characterization of Hydrophobic Stilbene Derivatives

##### 2.2.2.1 Proton Nuclear Magnetic Resonance

The chemical structure was analyzed using proton nuclear magnetic resonance (NMR-H) on a Bruker Avance 600 MHz spectrometer (Bruker, Germany) at 25 °C. Tubes of 3 mm containing 3 mg of pure compounds dissolved in 0.3 mL of deuterated chloroform were used for the analysis. Chemical shifts (δ) were expressed in ppm, and the difference between natural and modified stilbenes was calculated.

##### 2.2.2.2 Spectrophotometric Analysis

Serial dilutions were made at increasing concentrations of stilbenes from 1.56 to 400 μM in methanol. Then, their absorption spectra were recorded between 200 and 600 nm using a Jasco V-630 spectrophotometer (Madrid, Spain) with Thorlabs CV10Q1400 cuvettes, and the maximum absorbance wavelength (λ_max_) for each stilbene was determined. The extinction coefficient or absorptivity was calculated by means of Lambert–Beer’s law.

##### 2.2.2.3 Fluorescence Spectroscopy Analysis

Serial dilutions were made at increasing concentrations of stilbenes from 1.56 to 400 μM in methanol. Excitation spectra between 200 and 360 nm and emission spectra between 340 and 500 nm were recorded using a Shimadzu RF-6000 spectrofluorometer (Shimadzu, Kyoto, Japan) with thermostatically controlled cells, setting the excitation and emission bandwidths to 3 and 5 nm, respectively. The wavelength of maximum excitation (λ_ex_) and maximum emission (λ_em_) for each stilbene was determined, and it was used to measure their emission and excitation spectra, respectively.

##### 2.2.2.4 Prediction of Other Physicochemical Parameters

The determination of lipophilicity or octanol/water coefficient (logP) and aqueous solubility (logS) was performed using the ALOGPS software (version 2.1).^28^ The prediction accuracy of logP with this software has a root-mean-square error (RMSE) of 0.35 and a standard error of the mean (SEM) of 0.26, while the prediction accuracy of logS has an RMSE of 0.49 and an SEM of 0.38. The determination of the topological polar surface area (TPSA) and molecular volume was carried out using the Molinspiration software (versions 2018.10 and 2018.03) (Molinspiration Cheminformatics, Slovensky Grob, Slovakia).

#### 2.2.3 Synthesis of HBCD-Pols

HBCD-Pols were formed by the cross-linking of pyromellitic dianhydride (PMDA).^25,29^ This cross-linking was carried out with anhydrous DMSO and triethylamine in glass scintillation vials, adding β-CD until complete dissolution. Then, the required amount of PMDA was added to obtain a cyclodextrin and PMDA ratio of 1:12 and left to react for 24 h at room temperature. The resulting product was precipitated by adding ethyl acetate in excess. Subsequently, the sample was filtered with more ethyl acetate to remove impurities and dried. Then, it was dissolved in deionized water, lyophilized, and subjected to a Soxhlet extraction with acetone for 24 h to completely remove residues and unreacted reagents. The total removal of residual DMSO was verified by elemental analysis. The resulting white powder was dried, ground with a mortar and pestle, and stored in darkness.

#### 2.2.4 Cell Culture Studies

##### 2.2.4.1 Cell Line and Culture Conditions

The human colorectal Caco-2 cell line was obtained from the ATCC (American Type Culture Collection) (ATCC, Rockville, USA), and the supplier’s recommendations for use were followed. Cells were cultured in complete EMEM without phenol red, with 10% (v/v) FBS, 2 mM l-glutamine, and 1% (v/v) penicillin/streptomycin. The cell line was maintained in an incubator at 37 °C with 5% CO_2_ and 85% relative humidity.

##### 2.2.4.2 Cell Permeability and Metabolism

The Caco-2 cell monolayer was formed by seeding 260,000 viable cells/cm^2^ onto 3 μm polycarbonate inserts incorporated into 24-well Costar Transwell plates from Corning (Corning, NY, USA). The apical chamber (insert) was filled with the medium up to 140 μL, and the basolateral chamber (well) was filled up to 860 μL. Cells were grown under usual culture conditions, with the integrity of the monolayer checked periodically by measuring its transepithelial electrical resistance (TEER) using a MillicellERS-2 voltmeter.^30^ Once reaching a value equal to or greater than 300 Ω·cm^2^ considering the inherent resistance of the inset membrane, the cell permeability assay was initiated. Experiments were conducted with an apical → basolateral flow, so treatments were added to the inset chamber, and aliquots were collected from the well chamber. Sink conditions were maintained through the experiment.

On the day of the assay, the medium was removed from the cells, and they were preincubated under usual culture conditions with complete HBSS (with 25 mM HEPES) for 15 min with gentle orbital agitation. After this time, the medium was removed, 600 μL of complete HBSS was added to the basolateral chamber, and 100 μL of complete HBSS supplemented with the compounds of interest was added to the apical chamber. The concentration of stilbenes was 300 μM, and HBCD-Pols were added to enhance solubilization at 100 ppm based on previous studies.^29^ The concentration of DMSO applied with the treatments did not exceed 1% (v/v) of the final volume based on previous studies.^31,32^ All treatments were previously sterilized using 0.2 μm sterile filters or UV light (for HBCD-Pols). Cell-free blanks were used to confirm that the compounds passed through the inset filter.

Aliquots of 300 μL were collected from the basolateral chamber at 0, 0.5, 1, 1.5, 2, 4, 6, and/or 24 h, with this volume replaced with the medium used during the experiment. Additionally, aliquots were collected from the apical chamber at the beginning and end of the experiment. Monolayer integrity was verified at the end of the assay by measuring TEER using a MillicellERS-2 voltmeter. All collected samples were stored at −20 °C and processed as soon as possible. For cellular metabolism studies, the total content of sulfate and glucuronide metabolites in the apical and basolateral regions was analyzed after 24 h.

The apparent permeability coefficient (*P*_ap_) was determined from the concentration versus time data using the following equation:^33^

Pap(cms)=dcdt·VA·c0 1

where (*dc/dt*) is the steady-state flux (μM/s), *V* is the volume in the basolateral chamber (mL), *A* is the area of the inset membrane (cm^2^), and *c*_0_ is the initial concentration in the apical chamber (μM).

##### 2.2.4.3 Extraction of Stilbenes from Extracellular Media

The extraction of the compounds of interest from cellular media was carried out by mixing with acetonitrile in a 1:1 (v/v) ratio. This mixture was then vortexed and centrifuged at 13,000 rpm for 10 min. The supernatants containing the compounds of interest were recovered and evaporated. Finally, the evaporated samples were resuspended in methanol and injected into an HPLC system, along with the corresponding calibration curve for each compound to be analyzed, except for sulfate and glucuronide metabolites, which were estimated by their *m*/*z* ratio. An Agilent HPLC series 1200 equipped with a TOF 6220 detector (acquisition range 100–1100) in negative mode was used, with a Kromasil C18 column of 5 μm (150 × 4.6 mm) (Phenomenex, Torrance, CA, USA) and a flow rate of 0.8 mL/min at 25 °C. The method used was an aqueous gradient of methanol (with 20 mM ammonium acetate): 30% from 0 to 5 min, 40% from 8 to 9.5 min, 50% at 12 min, 99% from 12.5 to 25 min, and 30% from 25.01 to 30 min.

#### 2.2.5 Data Analysis

All experiments were conducted at least in triplicate, increasing the number of replicates per assay in the case of *in cellulo* experiments. Graphical representations were performed using SigmaPlot (version 10.0.0.54). The results were statistically analyzed setting a significance value of *p* < 0.05 using the *t* test with the Rstudio software (version 0.99.878).

## 3 Results and Discussion

### 3.1 Efficiency of the Synthesis of Resveratrol Stearate by Chemical and Enzymatic Approaches

Resveratrol stearate was synthesized by chemical and enzymatic reactions to compare both methodologies and select the optimal for the synthesis of all the hydrophobic stilbene derivatives. Both reactions were very specific in the formation of stilbene monoesters (Figure 1), with minimal diester formation in the chemical synthesis, which could not be detected in the enzymatic synthesis.

**Figure 1:**
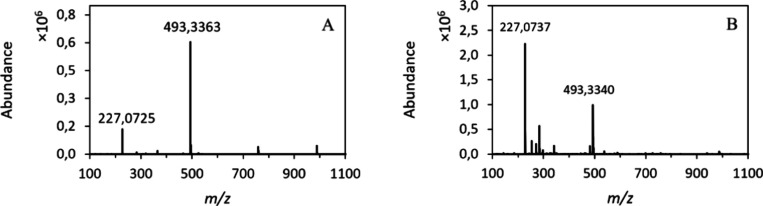
Mass spectrum ESI(−) of the (A) chemical synthesis and (B) enzymatic synthesis of resveratrol stearate (*m*/*z* 493) using resveratrol (*m*/*z* 227) as a substrate

The chemical synthesis of resveratrol stearate achieved a yield of 72% after 24 h (Figure 1A), while the enzymatic synthesis achieved a maximum yield of 56% after 90 h of incubation with the Novozym 435 enzyme (Figure 1B). At the end of the enzymatic reaction, vinyl stearate was transformed into stearic acid, and there was still a considerable amount of unreacted vinyl stearate and resveratrol. Meanwhile, the amount of unreacted substrates in the chemical reaction was much lower than that in the enzymatic reaction.

In a previous study in which resveratrol dodecanoate was synthesized chemically and enzymatically,^34^ the authors also obtained higher yields with the chemical method (25–40%) compared to the enzymatic method (9–15%).

The yields obtained in this work show that the chemical synthesis and enzymatic synthesis developed are quite efficient and selective in the formation of monoesters in comparison with previous literature reports on similar esterification reactions with other substrates and/or catalysts. Regarding chemical synthesis, Tain et al. (2020) obtained a similar yield of 73% in the synthesis of resveratrol butyrate with a Steglich esterification using *N*-ethyl-*N*′-(3-(dimethylamino)­propyl)-carbodiimide (EDC) instead of DCC and THF as solvent.^35^ Oh and Shahidi (2017) synthesized several hydrophobic resveratrol derivatives using chlorinated fatty acid derivatives as substrates. With this process, they achieved yields of 37.7% resveratrol docosahexanoate and 74% resveratrol caprylate but also obtained notable amounts of diesters and triesters.^36^ Pany et al. (2012) obtained similar yields to those of this work for the synthesis of resveratrol oleate (77%), resveratrol linoleate (70%), and resveratrol linolenate (68%).^37^ The process followed by these authors achieved a single isomer modified at the 4′-OH group but was also a more complex method as it premethylated the 3′-OH and 5′-OH groups before to achieve this regioselectivity. Wong et al. (2020) achieved yields of 23 – 78% by conjugating the stilbene combretastatin A4 and some isomers with stearic, oleic, linoleic and linolenic acid.^38^

In relation to enzymatic synthesis, Torres et al. (2010) obtained a maximum yield of 35% of resveratrol stearate after 160 h of reaction using a higher proportion of enzyme and vinyl stearate than the one optimized in this work.^39^ The same authors carried out a similar reaction using vinyl acetate as a substrate and achieved a much higher yield (80%) of resveratrol acetate after 50 h. Nicolosi et al. (2002) and Teng et al. (2005) also synthesized resveratrol acetate with Novozym 435 using different substrate ratios but achieved lower yields of 40% after 90 h and 45% after 72 h, respectively.^27,40^ All these authors emphasized that the process was very selective, yielding a single monoester product modified at the 4′-OH group, so it is likely that the resveratrol stearate synthesized in this work with the same enzyme also shows such regioselectivity.

### 3.2 Synthesis and Purification of Hydrophobic Stilbene Derivatives

Since the chemical synthesis gave a higher yield in much less time and without the need for high temperatures, this procedure was selected for the synthesis of the other hydrophobic stilbene derivatives. Several reactions were performed to obtain resveratrol stearate (RE, *m*/*z* 493), resveratrol oleate (RO, *m*/*z* 491), resveratrol palmitate (RP, *m*/*z* 465), oxyresveratrol stearate (OE, *m*/*z* 509), oxyresveratrol oleate (OO, *m*/*z* 507), oxyresveratrol palmitate (OP, *m*/*z* 481), piceatannol stearate (PcE, *m*/*z* 509), piceatannol oleate (PcO, *m*/*z* 507), piceatannol palmitate (PcP, *m*/*z* 481), pterostilbene stearate (PtE), pterostilbene oleate (PtO), and pterostilbene palmitate (PtP) products (Figure 2).

**Figure 2:**
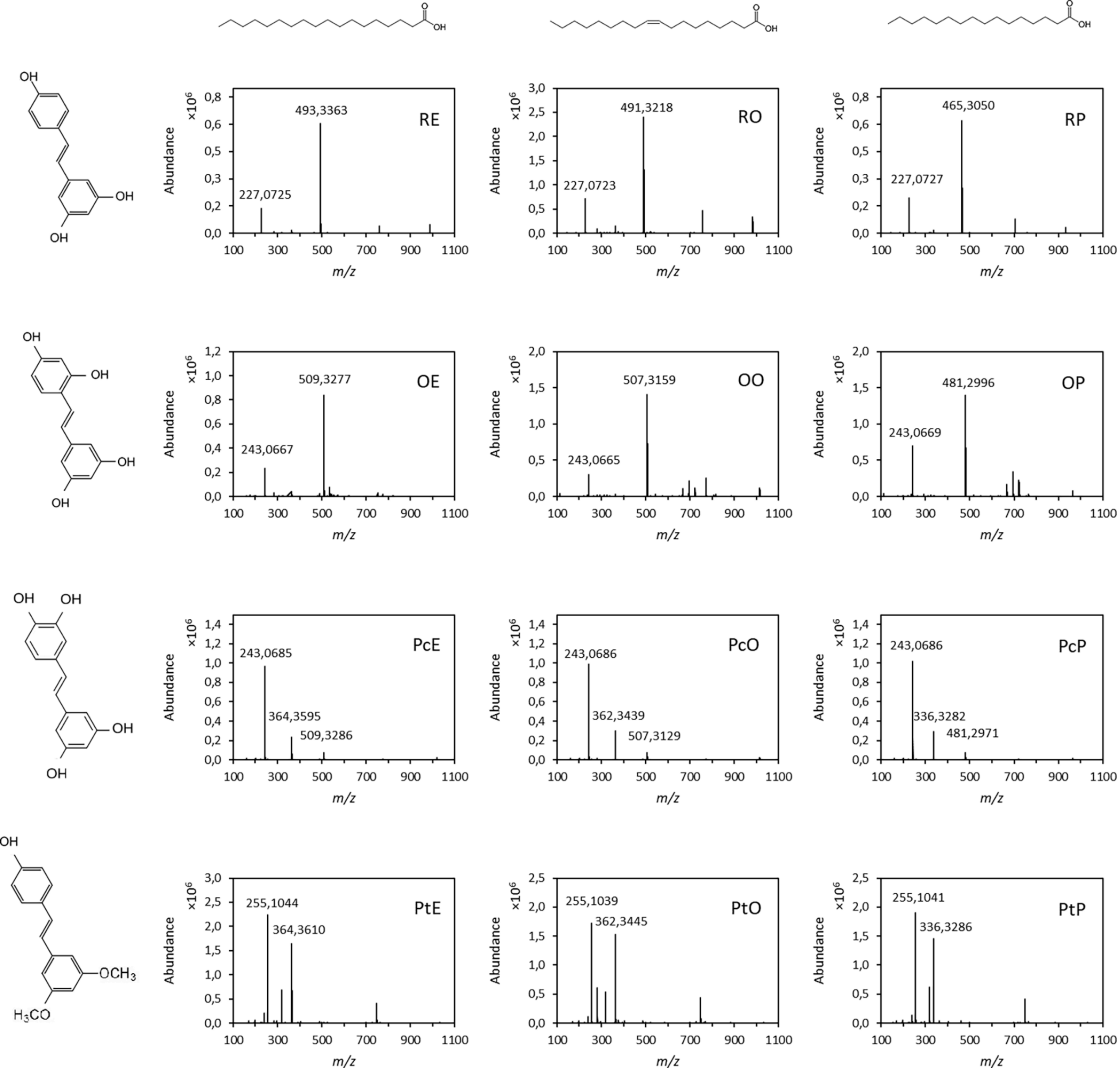
Mass spectra ESI(−) of chemical synthesis reactions of hydrophobic stilbene derivatives. For each reaction, the structures of the substrates used are displayed above and on the left.

From the 12 different chemical synthesis reactions, a total of 21 monoester products that can be detected by liquid chromatography were obtained, including some positional isomers.

The yields of the reactions with resveratrol and oxyresveratrol determined by mass spectrometry were 61–76% and 49–78%, respectively (Table 1). Yields could not be properly determined for the reactions with pterostilbene and piceatannol due to the difficulty to ionize and ease of degradation by breaking the ester bond, respectively. Despite this, these reactions proved to be less efficient as the formation of a secondary product was observed, which was subsequently identified as a potential DCC derivative with the fatty acids used (*m*/*z* 364, 362, and 336 with stearic acid, oleic acid, and palmitic acid, respectively) (Figure 2).

**Table 1:** Yields and Isomeric Products in the Chemical Synthesis of Hydrophobic Stilbene Derivatives^a^

**product**	**isomer**	**formula**	**MW**	*m*/*z* ^–^	**yield (%)** HPLC-ESI(−)	**isomer ratio (%)** HPLC-DAD
RE	RE i1	C_32_H_46_O_4_	494.7052	493.3363	72	28
	RE i2					72
RO	RO i1	C_32_H_44_O_4_	492.6894	491.3218	76	32
	RO i2					68
RP	RP i1	C_30_H_42_O_4_	466.6521	465.3050	61	32
	RP i2					68
OE	OE i3	C_32_H_46_O_5_	510.7046	509.3277	74	80
	OE i1					8
	OE i2					12
OO	OO i3	C_32_H_44_O_5_	508.6888	507.3159	78	77
	OO i1					10
	OO i2					12
OP	OP i3	C_30_H_42_O_5_	482.6515	481.2996	49	75
	OP i1					13
	OP i2					12
PcE		C_32_H_46_O_5_	510.7046	509.3286	ND	100
PcO		C_32_H_44_O_5_	508.6888	507.3129	ND	100
PcP		C_30_H_42_O_5_	482.6515	481.2971	ND	100
PtE		C_34_H_50_O_4_	522.7584	NI	ND	100
PtO		C_34_H_48_O_4_	520.7425	NI	ND	100
PtP		C_32_H_46_O_4_	494.7052	NI	ND	100

a ND = not determined; NI = nonionizable.

The synthesized monoesters were purified by column chromatography with silica gel, yielding a racemic mixture of stable ratio in resveratrol and oxyresveratrol derivatives (Table 1). In resveratrol derivatives, two chromatographic peaks were observed, eluting close to each other and corresponding to two isomers (R- i1 and R- i2). Meanwhile, in oxyresveratrol derivatives, up to three chromatographic peaks were observed, two also eluting close together (O- i1 and O- i2) and a more separated major peak eluting first (O- i3), suggesting large differences in polarity depending on the modified group (Figure 3).

**Figure 3:**
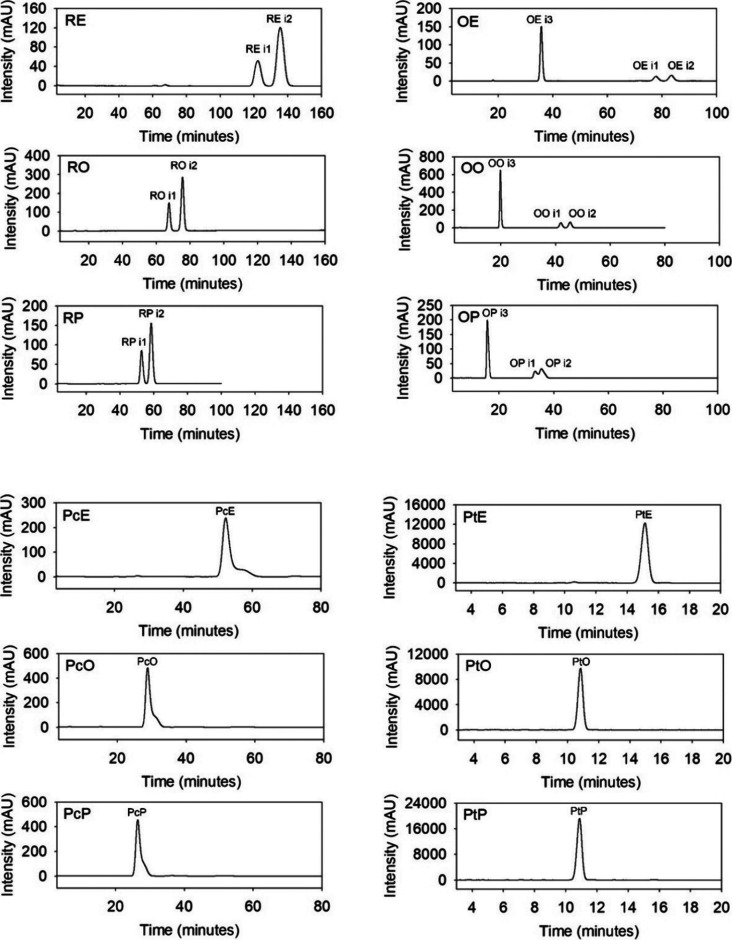
Chromatograms at 310 nm of the hydrophobic stilbene derivatives with an isocratic mobile phase consisting of 85% methanol for the resveratrol, oxyresveratrol, and piceatannol derivatives and 99% methanol for the pterostilbene derivatives.

The piceatannol derivatives showed a tail at the end of the chromatographic peaks, especially piceatannol stearate (Figure 3). Although this could indicate the presence of another isomer, a second peak could not be separated by varying the method, and no variation in the absorption spectrum between the peak and the tail was observed (data not shown).

As expected, pterostilbene derivatives revealed only one chromatographic peak corresponding to the esterification at the 4′-OH position. Since these products were pure isomers, the chemical shifts in the stilbene molecule were determined by H NMR. The results confirmed that the closest hydrogens at the 3′ and 5′ positions suffered a notable increase of approximately 0.250 ppm, unlike the hydrogens at the unmodified ring (positions 4, 2, 6, 3, and 5) that showed very low or even zero variation in chemical shifts (Table 2). The viscosity of pterostilbene oleate prevented it from being properly analyzed by this technique.

**Table 2:**
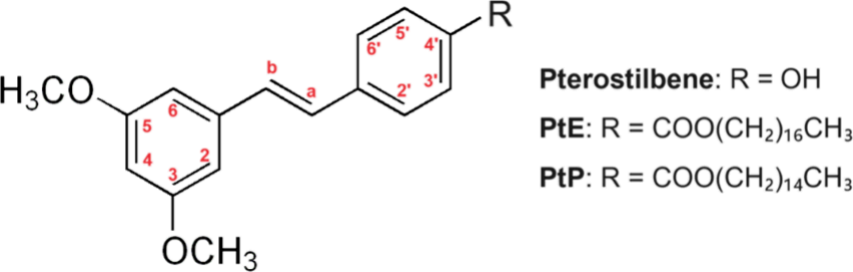
Chemical Shifts of Pterostilbene and Its Hydrophobic Derivatives by H NMR

	**chemical shift (ppm)**		
H position	**pterostilbene**	**PtE**	**PtP**
3, 5	3.813	3.812	3.813
4	6.366	6.380	6.380
2, 6	6.632	6.640	6.641
a	6.878	6.961	6.962
b	7.006	7.027	7.048
3′, 5′	6.808	7.054	7.059
2′, 6′	7.383	7.482	7.482

### 3.3 Characterization of Hydrophobic Stilbene Derivatives

The spectrophotometric, spectrofluorometric, and other physicochemical properties of the synthesized hydrophobic stilbene derivatives were analyzed to facilitate future research with these molecules.

The derivatization of stilbenes with fatty acids caused a modification in their absorption spectra, which varied according to the hydroxyl group that was modified. In general, the absorption spectra of the derivatives showed a very similar pattern to the original stilbenes (Figure S1) with changes in the λ_max_ (Table 3). The λ_max_ values were determined for the isomers separately and for the racemic mixture obtained, determining the molar absorptivity value for the racemic mixture of the hydrophobic derivatives (Table 3). Piceatannol derivatives could not be fully characterized because of their ease of degradation, breaking the ester bond and releasing piceatannol. The most highlighted change was observed for the major isomer in oxyresveratrol derivatives, whose shape showed only one peak with a hypsochromic shift of 22 nm compared to oxyresveratrol (Figure S1).

**Table 3:** Spectrophotometric and Spectrofluorimetric Characterization of Hydrophobic Stilbene Derivatives and Their Original Stilbenes^a^

**product**	**isomer**	**λ**_**max**_ **(nm)** HPLC-DAD	**λ**_**max**_ ^b^ **(nm)** UV/vis	**ε** ^b^ **(M** ^**–1**^ **cm** ^**–1**^ **)**	**λ**_**ex**_ ^b^ **(nm)**	**λ**_**em**_ ^b^ **(nm)**
resveratrol			306	30 257	304	377
RE	RE i1	320^c^	304	5 948	301	408
	RE i2	302^c^				
RO	RO i1	320^c^	304	19 620	301	407
	RO i2	302^c^				
RP	RP i1	320^c^	304	9 006	301	408
	RP i2	302^c^				
oxyresveratrol			328	27 044	327	393
OE	OE i3	306^c^	320	605	322	384
	OE i1	330^c^				
	OE i2	324^c^				
OO	OO i3	306^c^	320	3 931	320	384
	OO i1	332^c^				
	OO i2	322^c^				
OP	OP i3	306^c^	307	1 064	301	405
	OP i1	332^c^				
	OP i2	322^c^				
piceatannol			326	32 956	ND	ND
PcE		314^c^	ND	ND	ND	ND
PcO		312^c^	ND	ND	ND	ND
PcP		312^c^	ND	ND	ND	ND
pterostilbene			307	44 991	305	377
PtE		302^b^	302	8 149	300	385
PtO		302^b^	302	3 220	299	385
PtP		302^b^	302	8 929	299	385

a λ_max_ = maximum absorbance wavelength; ε = extinction coefficient or absorptivity; λ_ex_ = fluorescence maximum excitation wavelength; λ_em_= fluorescence maximum emission wavelength; ND = not determined.

b Measured in methanol.

c Measured in 85% methanol.

All hydrophobic stilbene derivatives decreased molar absorptivity compared to the original stilbenes (Table 3). These results seem to indicate that the addition of a hydrocarbon chain interacts with the ability of stilbenes to absorb electromagnetic radiation, resulting in lower absorptivity values, probably due to a breakdown of the resonance of the aromatic rings. Although the range of linear concentrations increases, these changes generate greater difficulty for the spectrophotometric quantification of these compounds.

In contrast to absorbance, derivatization with fatty acids seems to be able to enhance the fluorescent signal of resveratrol and pterostilbene derivatives, facilitating spectrofluorometric quantification of these hydrophobic stilbene derivatives (Figure 4).

**Figure 4:**
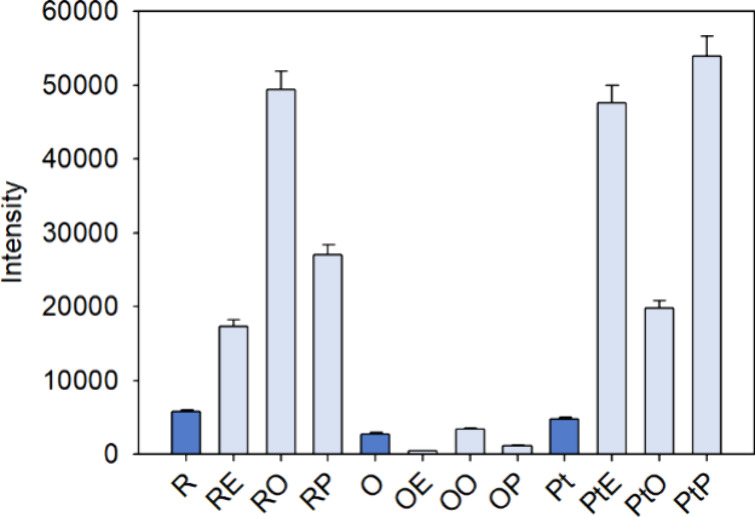
Fluorescence emission intensity of hydrophobic stilbene derivatives (light color) and their original stilbenes (dark color) at 100 μM. R: resveratrol, O: oxyresveratrol, Pt: pterostilbene.

Overall, the shapes of the fluorescence excitation and emission spectra were quite similar for the stilbenes before and after structural modification (Figures S2 and S3), with the main differences observed in the λ_em_ (Table 3). Compared to the original stilbenes, resveratrol derivatives showed a marked increase in λ_em_ by 31 to 32 nm, while pterostilbene derivatives showed a marked increase by 8 nm. In contrast, oxyresveratrol derivatives showed more differences in λ_em_ according to the fatty acid used in the esterification, increasing up to 15 nm for oxyresveratrol palmitate and decreasing by 9 nm for oxyresveratrol stearate and oleate.

Other physicochemical parameters were computationally determined (Table S4). As expected, this structural modification of stilbenes led to an increase in their lipophilicity (of 3 to 4 times higher approximately) accompanied by a decrease in their aqueous solubility (of 2 times lower approximately). Moreover, the logP values were well correlated with the chromatograms obtained above (Figure 3). This marked increase in lipophilicity could favor transport through the lymphatic system; nevertheless, *in vivo* studies are required to demonstrate this.

Based on the logP results, the identification of the isomeric products synthesized could be deduced, with the isomers named i1 being those modified in the 3-OH or 5-OH group, i2 those modified in the 4′-OH group, and i3 those modified in the 2′-OH group. However, more experimental studies are needed to confirm it.

### 3.4 Cell Permeability of Hydrophobic Stilbene Derivatives

The ability of the Caco-2 intestinal cell line to grow as a monolayer allows for *in vitro* assays that predict the absorption of orally administered bioactive compounds.^41^ Therefore, to investigate the uptake of the synthesized hydrophobic stilbene derivatives, their permeability across a monolayer of Caco-2 cells was evaluated.

Hydrophobic derivatives of resveratrol and oxyresveratrol were used for these analyses due to their overall good yield and their greater ease of quantification and solubilization. Moreover, to improve the solubilization of these derivatives in the medium during the tests, HBCD-Pols were added according to the previous literature.^25,29^ The integrity of the cell monolayer was checked by transepithelial electrical resistance measurements at both the beginning and end of the experiments.

The results showed that in the presence of HBCD-Pols, hydrophobic resveratrol derivatives improved the apparent permeability of resveratrol, while hydrophobic oxyresveratrol derivatives worsened the apparent permeability of oxyresveratrol (Figure 5A). Apart from the type of stilbene, the fatty acid used in the esterification also significantly influenced the ability of the derivative to cross the cell monolayer, with oleates showing the highest permeability followed by stearates and palmitates. Within the hydrophobic stilbene derivatives, resveratrol oleate gave the best results, even exceeding the high apparent permeability of oxyresveratrol. Resveratrol stearate, resveratrol palmitate, and oxyresveratrol oleate gave similar permeability, with no significant differences between them, all exceeding the apparent permeability of resveratrol.

**Figure 5:**
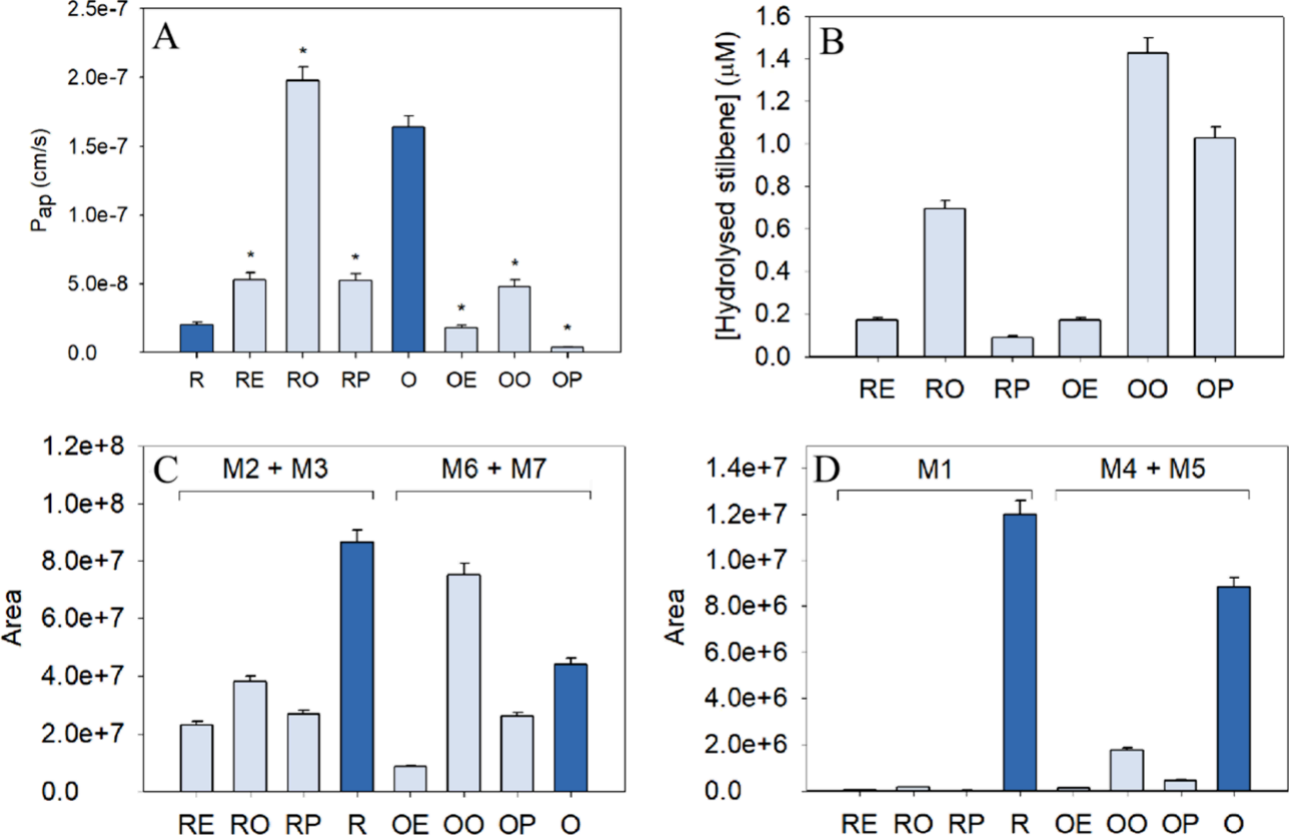
(A) Apparent permeability of hydrophobic stilbene derivatives (light color) and their natural stilbenes (dark color) in the presence of HBCD-Pols in the Caco-2 cell line calculated from eq 1. (B) Hydrolysis of hydrophobic stilbene derivatives by Caco-2 cells after 24 h determined by HPLC-ESI(−). Formation of (C) sulfate and (D) glucuronide metabolites of resveratrol or oxyresveratrol by Caco-2 cells after 24 h incubation (apical + basolateral) with hydrophobic stilbene derivatives (light color) and their original stilbenes (dark color) determined by HPLC-ESI(−).

There are no previous studies on the apparent permeability of hydrophobic stilbene derivatives such as the ones synthesized in this work. However, Thaweesest et al. (2022) analyzed other smaller derivatives, such as tetraacetate, tetrapropionate, and tetrabutyrate of oxyresveratrol, observing an increase in their apparent permeability compared to oxyresveratrol.^42^ The apparent permeability of resveratrol and oxyresveratrol has been previously determined, although under different conditions and without the presence of HBCD-Pols.^42–47^ In general, these studies obtained orders of magnitude of 10^–5^ to 10^–7^ using initial concentrations between 5 and 250 μM and incubation times between 1 and 16 h for an apical → basolateral transport. In this research, a lower apparent permeability with an order of magnitude of 10^–8^ was obtained using an initial concentration of 300 μM in the presence of 100 ppm of HBCD-Pols and incubation time of 24 h for apical → basolateral transport. Further apparent permeability studies using reference compounds are required to compare the permeabilities obtained with those of other drugs.

The lower permeability could be due to the presence of the HBCD-Pols that cause a controlled release of stilbenes, delaying their passage through the cell monolayer and thus reducing their apparent permeability during the experimental time.^48^ Differences in the interactions of the compounds with CD-based polymers could also influence their permeability.

Moreover, although the apparent permeability of the individual isomers was not determined, it could be observed that the hydrophobic oxyresveratrol derivatives varied significantly in their isomer ratio between the beginning and the end of the experiments, whereas the resveratrol derivatives remained almost the same (Table 4).

**Table 4:** Isomer Ratio at the Beginning (0 h) and at the End (24 h, Apical and Basolateral Average) of the Permeability Assays in the Presence and Absence of Caco-2 Cells, Determined by HPLC-ESI(−)^a^

		**isomer ratio (%)**		
			24 h	24 h
**product**	**isomer**	0 h	**with cells**	**without cells**
RE	RE i1	22	22	ND
	RE i2	78	78	ND
RO	RO i1	28	23	ND
	RO i2	72	77	ND
RP	RP i1	27	28	ND
	RP i2	73	72	ND
OE	OE i3	50	18	45
	OE i1	25	46	28
	OE i2	25	36	27
OO	OO i3	56	1	ND
	OO i1	20	19	ND
	OO i2	24	80	ND
OP	OP i3	67	1	ND
	OP i1	11	35	ND
	OP i2	22	64	ND

a ND = not determined.

In comparison with the isomer ratio previously obtained by the DAD detector, the isomer ratio obtained by the TOF detector varied slightly (Table 4), which could be due to a higher sensitivity of the mass spectrometer to detect the minor isomers of these derivatives. Despite this, the order of the isomers according to their proportion in the racemic mixture remained stable between the two detectors.

In all hydrophobic oxyresveratrol derivatives studied, the major isomer O- i3 became the minor isomer after 24 h of incubation with the intestinal cells, leaving at the end of the assay the O- i2 isomer as the major isomer in oxyresveratrol oleate and palmitate and the O- i1 isomer in oxyresveratrol stearate (Table 4). These changes could be due to an increased degradation or metabolization of the O- i3 isomer compared to the other isomers in the oxyresveratrol derivatives. To rule out a possible degradation due to the test medium or the test conditions, the ratio of oxyresveratrol stearate isomers was analyzed in the absence of the cells. These results showed smaller differences than those observed in the presence of cells (Table 4), so it is likely that this variation is due to a greater interaction of the O- i3 isomer with the intestinal cells.

### 3.5 Metabolism of Hydrophobic Stilbene Derivatives in Caco-2 Cells

The Caco-2 cell monolayer has a differentiated phenotype that shares many functions of the epithelium of the small intestine villi.^41^ Additionally, these cells express several functional enzymes related to phase II metabolism such as glucuronosyltransferases, glutathione-*S*-transferases, and sulfotransferases,^45^ enabling *in vitro* studies of the bioavailability and metabolism of bioactive compounds. For this reason, the formation of metabolites from phase II reactions in the Caco-2 cells after the treatment with resveratrol, oxyresveratrol, and their hydrophobic derivatives was investigated to better understand the interaction between these molecules and the intestinal cells. No conjugated metabolites of the esterified stilbenes were found in the extracellular medium of cells treated with the hydrophobic stilbene derivatives. However, it seems that some conjugated metabolites of their original stilbenes were present in these samples (Table 5).

**Table 5:** Metabolites in the Extracellular Media of Caco-2 Cells after 24 h (Apical + Basolateral) Incubation with the Hydrophobic Stilbene Derivatives or Their Original Stilbenes, Determined by HPLC-ESI(−)

						**isomer ratio (%)**							
**metabolite ID**		**formula**	**MW**	m/z^–^	**retention time (min)**	**RE**	**RO**	**RP**	**R**	**OE**	**OO**	**OP**	**O**
**M1**	resveratrol-*O*-glucuronide	C_20_H_20_O_9_	404.1098	403.1025	6.998	100	100	100	100				
**M2**	resveratrol sulfate	C_14_H_12_O_6_S	308.0356	307.0284	9.634	5	5	3	11				
**M3**					10.896	95	95	97	89				
**M4**	oxyresveratrol-*O*-glucuronide	C_20_H_20_O_10_	420.1047	419.0974	4.314					5	15	21	69
**M5**					6.659					95	85	79	31
**M6**	oxyresveratrol sulfate	C_14_H_12_O_7_S	324.0303	323.0231	7.846					1	0	1	1
**M7**					8.713					99	100	99	99

After 24 h of incubation, the extracellular media of cells treated with hydrophobic resveratrol and oxyresveratrol derivatives showed less formation of total sulfate (Figure 5C) and total glucuronide (Figure 5D) metabolites than the media of cells treated with the original stilbenes, except for the media with oxyresveratrol oleate, in which sulfate synthesis was higher than in the media with oxyresveratrol (Figure 5C). In particular, sulfate formation decreased between 2- and 5-fold in the derivatives compared to natural stilbenes, with the exception of oxyresveratrol oleate (Figure 5C), while glucuronide formation decreased 5-fold in oxyresveratrol oleate and more than 20-fold in the other derivatives, especially in resveratrol palmitate and resveratrol stearate (Figure 5D).

Although in the media of cells treated with resveratrol the formation of conjugated metabolites was higher than in the media of cells treated with oxyresveratrol, in general, the opposite occurred in the media treated with hydrophobic derivatives, with the hydrophobic resveratrol derivatives giving a lower formation of these metabolites compared to the hydrophobic oxyresveratrol derivatives (Figure 5C,D).

Different unidentified isomers of some of these metabolites could be detected according to the conjugated hydroxyl group, with a variable ratio depending on the amount of stilbene added to the medium. For hydrophobic resveratrol derivatives and resveratrol itself, the sulfate conjugate isomer eluting after 10 min of analysis, M3, was the major isomer, with a proportion of 89% (resveratrol treatment) and 95% or higher (hydrophobic resveratrol derivatives treatment) compared to the isomer with the shortest retention time, M2 (Table 5). A minimal part of the isomer of the oxyresveratrol sulfate conjugate with the shortest retention time, M6, was produced, accounting for only 1% or less of the total (Table 5). However, in the glucuronide conjugates of oxyresveratrol, the proportion of the isomeric metabolites varied markedly between the derivatives and oxyresveratrol. Meanwhile, in the derivatives, the glucuronide isomer M5 was the major isomer with a ratio of 79–95% to the M4 isomer; in oxyresveratrol, this M5 isomer with the longest retention time was the minor isomer, accounting for 31% of the total (Table 5). Previous studies on the intestinal metabolism of these natural stilbenes identified conjugates of resveratrol in the 3-OH or 4′-OH groups^49^ and of oxyresveratrol mainly in the 2-OH group.^50^

The hydrophobic derivatives of resveratrol and oxyresveratrol also showed some degree of hydrolysis in the extracellular media with concentrations of less than 1.5 μM of the original stilbenes that did not suffer from phase II reactions. Oxyresveratrol oleate was the derivative with the highest degree of hydrolysis followed by oxyresveratrol palmitate and resveratrol oleate (Figure 5B). In contrast, resveratrol palmitate showed the lowest degree of hydrolysis, forming only 90 nM resveratrol (Figure 5B).

The conjugation of stilbenes during metabolism after oral administration is responsible for the low bioavailability of these bioactive compounds.^51^ The results of this research show that despite the slight level of hydrolysis observed, hydrophobic stilbene derivatives seem to have a greater ability to maintain the aglycone form than natural stilbenes. This may be due to the masking of free hydroxyl groups with fatty acids, which prevents phase II enzymes from acting on the stilbene. Therefore, the use of hydrophobic stilbene derivatives as stilbene precursors could help to avoid such metabolization reactions that negatively affect the bioavailability of stilbenes, supporting the first mechanism described above in the introduction. Further studies in whole organisms are required to determine whether the transport of these derivatives through the lymphatic system is another mechanism involved in the enhancement of their bioavailability, as well as to assess the possible biotransformation of these new derivatives by the gut microbiota, leading to the formation of other metabolites that may influence biological activity *in vivo*. Previous studies with other molecules also showed that the use of a more hydrophobic derivative can modify interactions with metabolic enzymes and aid the distribution of the molecule even though slight hydrolysis may occur during this process.^17,19,21^

In conclusion, hydrophobic derivatives of resveratrol, oxyresveratrol, piceatannol, and pterostilbene were synthesized with yields of up to 78% and high specificity in the formation of monoester products. The chemical synthesis was found to be more effective than the enzymatic synthesis. This structural modification of stilbenes led to changes in their physicochemical properties. It highlights that these new compounds might be more easily detectable by fluorometric than photometric techniques. Hydrophobic derivatives of resveratrol showed a higher apparent permeability across intestinal Caco-2 cells than resveratrol. This outcome was achieved in the presence of HBCD-Pol as a solubility enhancer; therefore, their influence on permeability must also be taken into account. Further apparent permeability studies using reference compounds are required to compare the permeabilities obtained with those of other drugs. Overall, hydrophobic derivatives of resveratrol and oxyresveratrol were more capable of retaining their aglycone form compared to their original stilbenes by forming fewer sulfate and glucuronide conjugated metabolites. This greater resistance to metabolism may be an advantage in avoiding phase II reactions that decrease the bioavailability of stilbenes. Therefore, hydrophobic stilbene derivatives could act as precursors of natural stilbenes with promising applications in the delivery of these bioactive compounds. Future studies in whole organisms could help to confirm the transport mechanisms of these new derivatives as well as other possible biotransformations following their oral intake.

## Supplementary Material





## Notes

Mass spectra of the reactions (ZIP)
Additional information on the spectra and other physicochemical properties of the hydrophobic stilbene derivatives (PDF)
