# Supplementary material for: Synthesis of Hydrophobic Derivatives of Stilbenes with Improved Permeability and Limited Phase II Reactions
Source: J Agric Food Chem. 2026 Apr 15;74(16):12945–55. doi: 10.1021/acs.jafc.6c00922 (PMC13133912; doi:10.1021/acs.jafc.6c00922)
Supplement: Supplementary file 2 [file jf6c00922_si_002.pdf]

Supporting Information

SYNTHESIS OF HYDROPHOBIC DERIVATIVES OF STILBENES WITH IMPROVED PERMEABILITY AND LIMITED PHASE II REACTIONS

Silvia Navarro-Orcajada[1*], Irene Conesa[1], Francisco José Vidal-Sánchez[1], Adrián Matencio[1,2], José Manuel López-Nicolás[1*]

[1] Departamento de Bioquímica y Biología Molecular-A, Facultad de Biología, Universidad de Murcia — Regional Campus of International Excellence "*Campus Mare Nostrum*", E-30100 Murcia, Spain

[2] Department of Chemistry, University of Turin, via P. Giuria 7, 10125 Turin, Italy

* Corresponding author: Tel: 34 868 884777 Fax: 34 868 364147

E-mail: josemln@um.es, silvia.navarro6@um.es

Figure S1. Relative absorption spectra of the hydrophobic stilbene derivatives (black) and their original stilbenes (blue). R: resveratrol, O: oxyresveratrol, Pc: piceatannol, Pt: pterostilbene

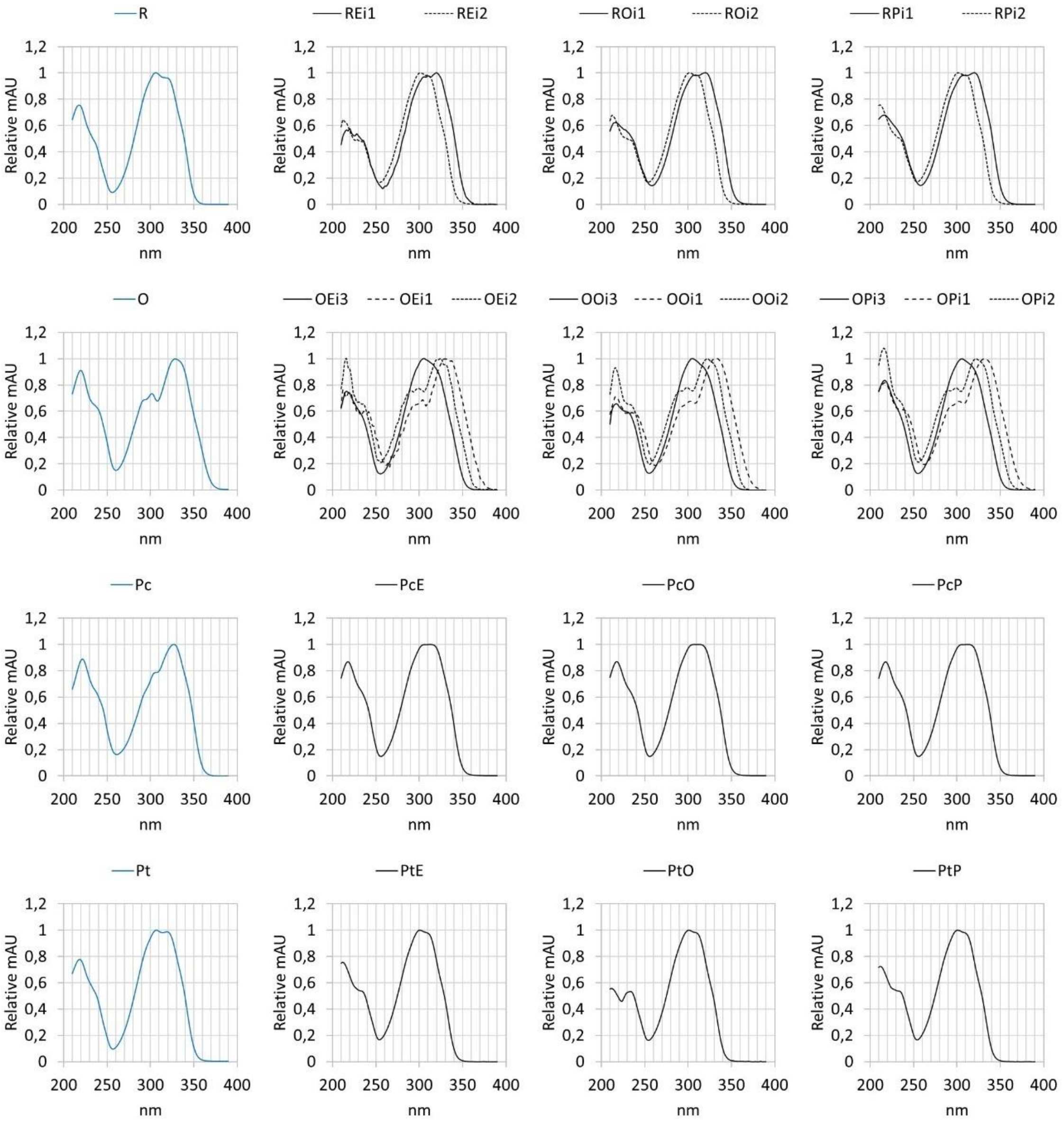

Figure S2. Relative fluorescence excitation spectra of the hydrophobic stilbene derivatives (black) and their original stilbenes (blue). R: resveratrol, O: oxyresveratrol, Pt: pterostilbene

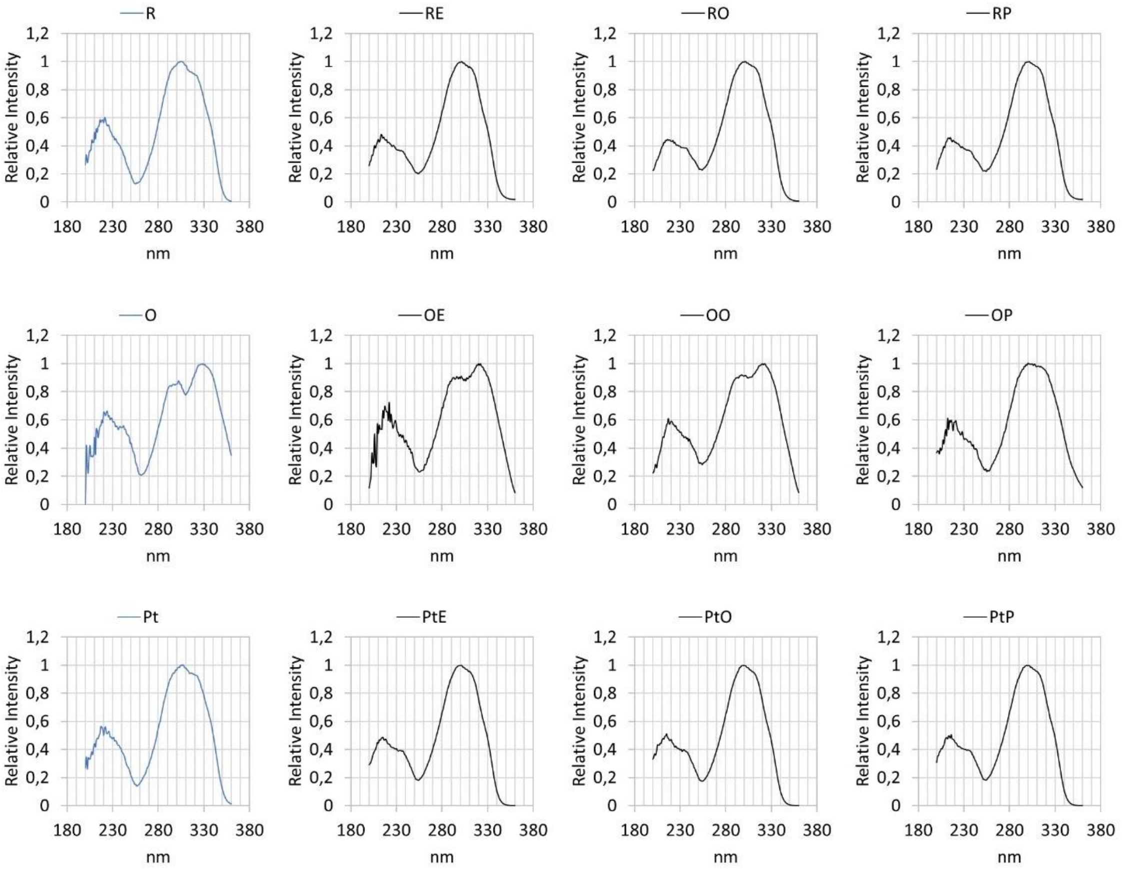

Figure S3. Relative fluorescence emission spectra of the hydrophobic stilbene derivatives (black) and their original stilbenes (blue). R: resveratrol, O: oxyresveratrol, Pt: pterostilbene

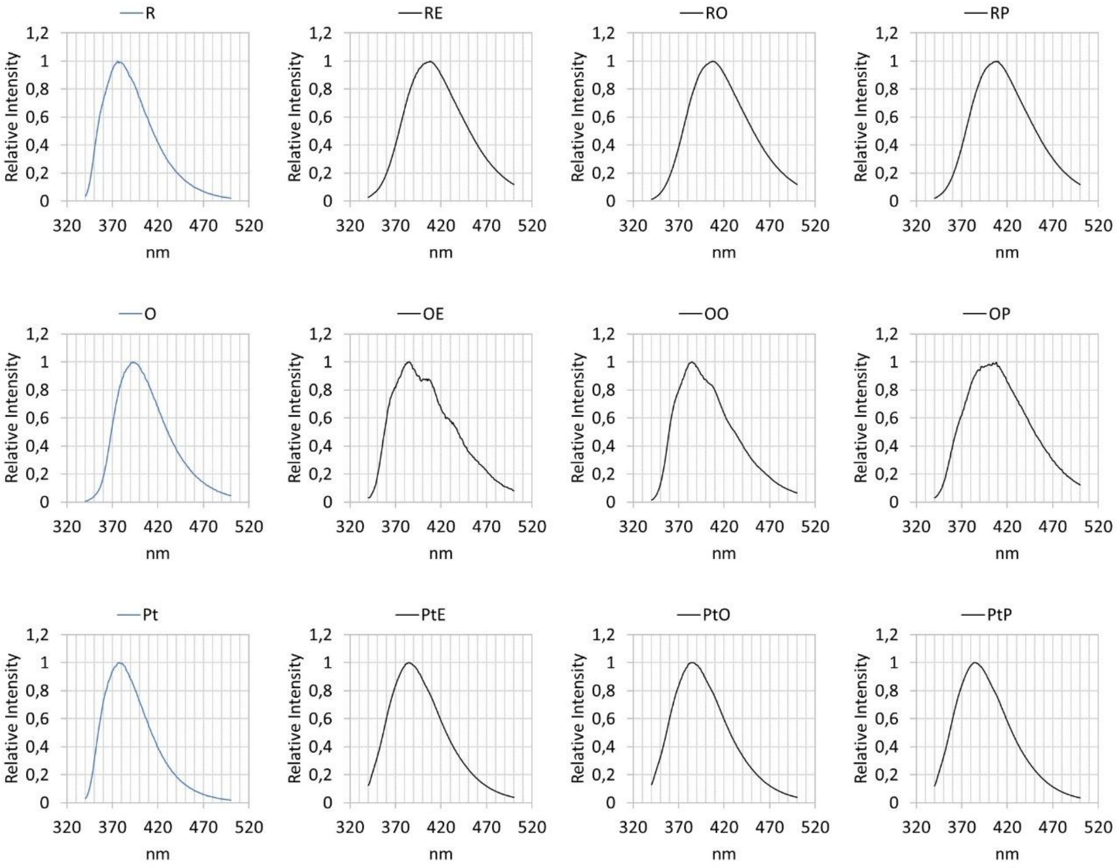

Table S4. Physicochemical parameters of hydrophobic stilbene derivatives and their original stilbenes. R: resveratrol, O: oxyresveratrol, Pc: piceatannol, Pt: pterostilbene

Product	Modified radical	-O-	-OH	logP	logS	TPSA ($Å^2$)	Molecular volume ($Å^3$)	MW
R	-	3	3	2.57	-3.52	60.68	206.92	228.2433
RE	R3 o R5	4	2	9.30	-7.04	66.76	512.26	494.7052
	R4’	4	2	9.34	-7.04	66.76	512.26	494.7052
RO	R3 o R5	4	2	9.07	-6.89	66.76	506.08	492.6894
	R4’	4	2	9.13	-6.90	66.76	506.08	492.6894
RP	R3 o R5	4	2	8.72	-6.75	66.76	478.66	466.6521
	R4’	4	2	8.80	-6.76	66.76	478.66	466.6521
O	-	4	4	2.06	-3.38	80.91	214.94	244.2427
OE	R2’	5	3	8.69	-6.43	86.99	520.28	510.7046
	R3 o R5	5	3	8.70	-6.43	86.99	520.28	510.7046
	R4’	5	3	8.72	-6.43	86.99	520.28	510.7046
OO	R2’	5	3	8.63	-6.39	86.99	514.09	508.6888
	R3 o R5	5	3	8.64	-6.39	86.99	514.09	508.6888
	R4’	5	3	8.66	-6.39	86.99	514.09	508.6888
OP	R2’	5	3	8.16	-6.15	86.99	486.68	482.6515
	R3 o R5	5	3	8.18	-6.15	86.99	486.68	482.6515
	R4’	5	3	8.19	-6.15	86.99	486.68	482.6515
Pc	-	4	4	2.12	-3.40	80.91	214.94	244.2427
PcE	R3’	5	3	8.70	-6.44	86.99	520.28	510.7046
	R4’	5	3	8.71	-6.43	86.99	520.28	510.7046
	R3 o R5	5	3	8.74	-6.45	86.99	520.28	510.7046
PcO	R3’	5	3	8.63	-6.39	86.99	514.09	508.6888
	R4’	5	3	8.64	-6.39	86.99	514.09	508.6888
	R3 o R5	5	3	8.69	-6.41	86.99	514.09	508.6888
PcP	R3’	5	3	8.18	-6.15	86.99	486.68	482.6515
	R4’	5	3	8.19	-6.16	86.99	486.68	482.6515
	R3 o R5	5	3	8.21	-6.17	86.99	486.68	482.6515
Pt	-	3	1	3.48	-4.37	38.70	241.98	256.2964
PtE	R4’	4	0	10.37	-8.11	44.77	547.32	522.7584
PtO	R4’	4	0	9.97	-8.18	44.77	541.13	520.7425
PtP	R4’	4	0	10.10	-8.15	44.77	513.72	494.7052
